# Divergent habitat use of two urban lizard species

**DOI:** 10.1002/ece3.3600

**Published:** 2017-11-23

**Authors:** Kristin M. Winchell, Elizabeth J. Carlen, Alberto R. Puente‐Rolón, Liam J. Revell

**Affiliations:** ^1^ Department of Biology University of Massachusetts Boston Boston MA USA; ^2^ Department of Biology Fordham University Bronx NY USA; ^3^ Departamento de Biología Universidad de Puerto Rico Mayagüez PR USA; ^4^ Programa de Biología Universidad del Rosario Bogotá Colombia

**Keywords:** anole, *Anolis cristatellus*, *Anolis stratulus*, niche partitioning, Puerto Rico, urbanization

## Abstract

Faunal responses to anthropogenic habitat modification represent an important aspect of global change. In Puerto Rico, two species of arboreal lizard, *Anolis cristatellus* and *A. stratulus*, are commonly encountered in urban areas, yet seem to use the urban habitat in different ways. In this study, we quantified differences in habitat use between these two species in an urban setting. For each species, we measured habitat use and preference, and the niche space of each taxon, with respect to manmade features of the urban environment. To measure niche space of these species in an urban environment, we collected data from a total of six urban sites across four different municipalities on the island of Puerto Rico. We quantified relative abundance of both species, their habitat use, and the available habitat in the environment to measure both microhabitat preference in an urban setting, as well as niche partitioning between the two different lizards. Overall, we found that the two species utilize different portions of the urban habitat. *Anolis stratulus* tends to use more “natural” portions of the urban environment (i.e., trees and other cultivated vegetation), whereas *A. cristatellus* more frequently uses anthropogenic structures. We also found that aspects of habitat discrimination in urban areas mirror a pattern measured in prior studies for forested sites in which *A. stratulus* was found to perch higher than *A. cristatellus* and preferred lower temperatures and greater canopy cover. In our study, we found that the multivariate niche space occupied by *A. stratulus* did not differ from the available niche space in natural portions of the urban environment and in turn represented a subset of the niche space occupied by *A. cristatellus*. The unique niche space occupied by *A. cristatellus* corresponds to manmade aspects of the urban environment generally not utilized by *A. stratulus*. Our results demonstrate that some species are merely tolerant of urbanization while others utilize urban habitats in novel ways. This finding has implications for long‐term persistence in urban habitats and suggests that loss of natural habitat elements may lead to nonrandom species extirpations as urbanization intensifies.

## 1 INTRODUCTION

Urbanization is one of the greatest sources of habitat change in the modern era. Urban areas occupy a large and expanding fraction of the landscape worldwide and are expected to increase in extent and intensity in coming years (Forman, 2014; United Nations 2012). Which species can tolerate this urbanization and how they achieve that end is an increasingly important aspect of ecology in an era of global change.

A major consequence of urbanization is the filtering of species, often resulting in lower biodiversity such that a subset of the original native community is represented in the urban habitat (Aronson et al., 2014; Forman, 2014). In general, urban communities are dominated by urban‐tolerant species, with abundances declining as urbanization increases and diversity declining as cities age (Forman, 2014; Grant, Middendorf, Colgan, Ahmad, & Vogel, 2011; McKinney, 2008; Shochat, Warren, Faeth, McIntyre, & Hope, 2006). However, urbanization can generate novel habitat space under some circumstances, resulting in new ecological opportunities and species colonization. While this may lead to enhanced abundance and diversity, these additions typically consist of nonnative species (McKinney, 2002; Shochat et al., 2006). Nonetheless, urban areas can still support substantial biodiversity (Forman, 2014).

Some species are able to adapt to changes associated with urbanization (e.g., Harris, Munshi‐South, Obergfell, & O'Neill, 2013; Winchell, Reynolds, Prado‐Irwin, Puente‐Rolón, & Revell, 2016). Species that persist in urban habitats tend to have several characteristics in common: broad habitat and diet requirements (generalists), high mobility, large reproductive output, small body size, and tolerance of human disturbances (Grant et al., 2011). Yet differences in tolerances and preferences between species mean that not all species are able to similarly meet their needs in urban environments.

Animals that persist in urban areas face a modified habitat dominated by human structures, lacking continuous canopy cover, and exhibiting different thermal and hydrologic conditions from those of natural areas nearby (reviewed in Forman, 2014). Some species persist in urban areas but are still dependent on nearby natural areas to maintain positive population growth. Such species are often referred to as “tolerant” or “urban adapters” (McKinney, 2002). By contrast, other species found in urban habitats have fully embraced their newfound milieu, utilize anthropogenic resources extensively, and may even achieve higher population growth rates and densities in urban areas than at natural sites. These species are referred to as “synanthropic,” “urbanophilic,” or “urban exploiters” (Forman, 2014; Grant et al., 2011; McKinney, 2006).

For urbanophiles, urbanization may have created a preferable habitat when compared to their historic natural area, with abundant food, refuges, and access to mates. For example, anthropogenic waste, along with insects attracted to this waste and to artificial light sources, may increase the availability of food on local scales for insectivorous herpetofauna (Henderson & Powell, 2001; Perry, Buchanan, Fisher, Salmon, & Wise, 2008). Shochat et al. (2010) found that elevated densities of some urban bird species can be attributed to both bottom‐up (more food resources) and top‐down (relaxed predation) controls. Urbanophilic species not only persist in urban habitats but also take full advantage of the novel environment and its resources, effectively expanding into the new niche space that can be associated with manmade structures and resources.

Puerto Rico provides an excellent opportunity to study animal responses to long‐term and intensifying human‐modification of the environment. Human settlement of the island by indigenous peoples began 5,000 years before the present day and was succeeded by intensive European settlement beginning in the early 16th century. These eras of colonization have resulted in successive periods of intense habitat modification. In particular, Puerto Rico was nearly completely deforested for the purposes of agriculture. This period of deforestation peaked in the 1940s when as little as 6% forest cover remained on the island (Koenig, 1953; Miller & Lugo, 2009). Over the past seven decades, forest cover has progressively regenerated as agricultural lifestyles were abandoned and industrialization increased, but this has been concurrent with an expansion and intensification of urban development island‐wide (Martinuzzi, Gould, & Ramos González, 2007; Miller & Lugo, 2009). The net result of this decline in agricultural and increase in industrialization has been a dramatic rise in the extent of urban areas by an estimated 42% in fewer than two decades (Helmer, 2004; López, Aide, & Thomlinson, 2001).

Presently, urban areas cover 11% of the island and continue to intensify in spatial extent and population density (Martinuzzi et al., 2007; Miller & Lugo, 2009; U.S. Census Bureau 2012). Urban areas are characterized by large percentages of impervious surfaces (e.g., roads, concrete) and low levels of vegetative canopy cover. With a population of 3,725,789 (93.8% of which reside in urban areas; U.S. Census Bureau 2012), Puerto Rico's urban areas include low‐density urbanization (such as rural communities), midsize cities dominated by suburban communities, and sprawling metropolitan areas with little to no remaining natural habitat and more than a million residents (e.g., Metropolitan San Juan). This intense land modification and the pressures associated with human presence have resulted in a decline of many native plant and animal species (Koenig, 1953; Miller & Lugo, 2009). Despite this centuries‐long process of urbanization, many native Puerto Rican species persist in and around urban sites.

**Figure 1:**
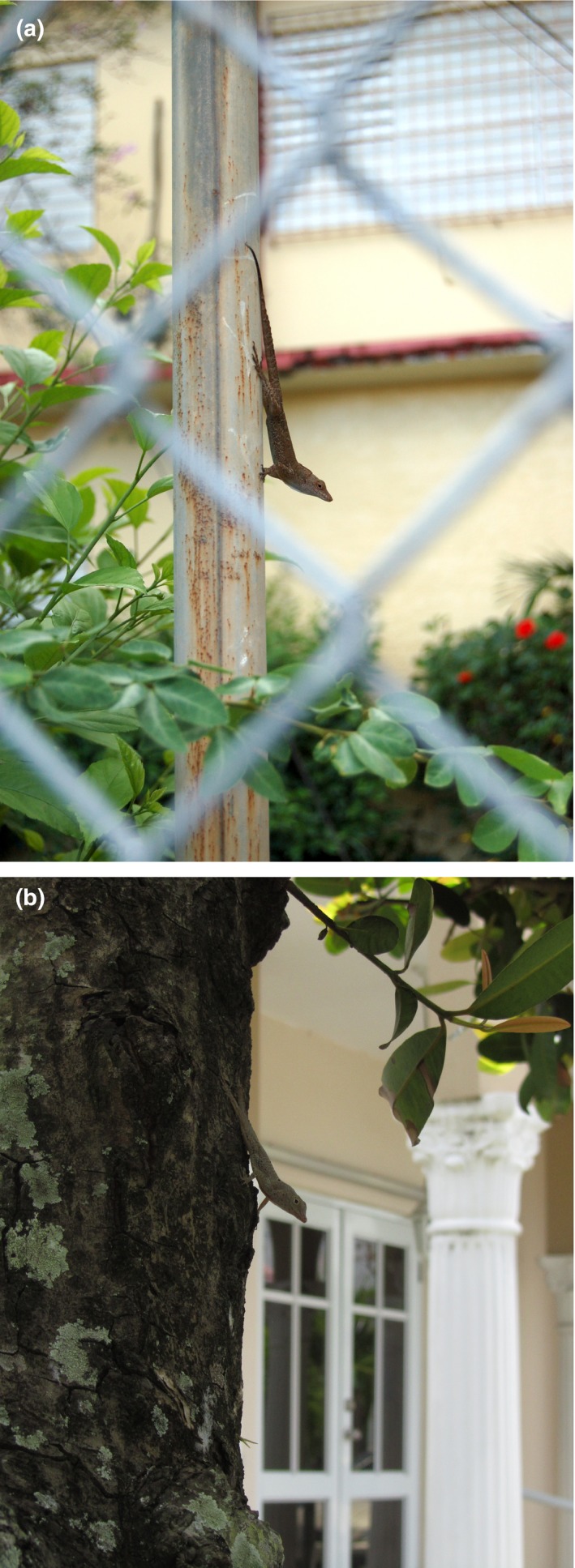
*Anolis cristatellus* (top; photo EJC) and *Anolis stratulus* (bottom; photo KMW) occupy different portions of the urban habitat

The Puerto Rican Crested Anole, *Anolis cristatellus*, and the Barred Anole, *Anolis stratulus* (Figure 1), are perhaps the most common urban anole species in Puerto Rico and are the only two we have regularly observed in virtually all types of urban areas island‐wide. Both species are relatively small arboreal lizards (adult male SVL 50–75 mm and 35–55 mm, respectively). Anoles are characterized by the repeated independent evolution of ecologically and morphologically similar microhabitat specialists (“ecomorphs”) on different islands. Among these, *Anolis cristatellus* is categorized as a “trunk‐ground” specialist whereas *A. stratulus* is a “trunk‐crown” specialist (Losos, 2009). These designations are defined by both habitat use and morphology. Trunk‐ground anoles, such as *A. cristatellus*, are brown in color, have relatively long limbs, a stocky build, and perch low to the ground on broad surfaces such as tree trunks. In contrast, trunk‐crown anoles, such as *A. stratulus*, are typically green in color (although *A. stratulus* is light gray), have relatively short limbs, are more slender, and perch from eye‐level to high up in trees, utilizing leaves, twigs, branches, and trunks as perches (reviewed in Losos, 2009).

*Anolis cristatellus* and *A. stratulus* are found at lower elevations and in both mesic and xeric habitats where they experience higher temperature and lower humidity compared to many upland species with more strict thermal requirements (Rand, 1964). Thus, it is perhaps unsurprising that both species are tolerant of urban habitat, which tends to be warmer and drier than nearby forests (reviewed in Forman, 2014). Where the two species co‐occur in natural forest habitats, they partition the habitat structurally and climatically. Specifically, prior research suggests that *A. stratulus* utilizes higher and thinner perches, and perch sites that are cooler, higher in humidity, and have more extensive canopy cover compared to habitat used by *A. cristatellus* (Rand, 1964; Reagan, 1992).

Here, we ask how these two species utilize and partition a novel habitat: urban areas. Our null hypothesis is that they will maintain niche partitioning and habitat choices in the same manner as they do in the natural forest habitats, choosing natural substrates that fit their preferences. In other words, *A. stratulus* will seek out higher, thinner, cooler perches with thicker canopy cover while *A. cristatellus* will seek out lower, broader, warmer perches with thinner canopy cover. Alternatively, both species may utilize the novel niche space associated with manmade habitat (i.e., on and around buildings). A third possibility is that only one species expands into the novel habitat space associated with anthropogenic resources while the other remains strictly associated with natural aspects of the urban space.

We tested these hypotheses by measuring the relative abundance, habitat use, and niche space occupied by these two species in urban areas. Because *A. cristatellus* is more of a generalist, we predict that it will occupy a greater portion of the urban habitat space and more extensively utilize habitat associated with anthropogenic structures while *A. stratulus* will remain associated with natural habitat elements. Similar to natural habitats, we also predict that each species will nonrandomly utilize perches that best meet their structural and climatic preferences as observed in natural areas. Understanding differential habitat use in urban persistent species sheds light on the factors influencing urban tolerance (the general capacity to persist in urban areas) versus genuine urbanophily. Here, we provide an empirical test of this ecological theory. Moreover, this type of natural history information can aid conservation in urban areas by identifying the minimum habitat requirements of native species and the potential for urban persistence and adaptation.

## 2 MATERIALS AND METHODS

**Figure 2:**
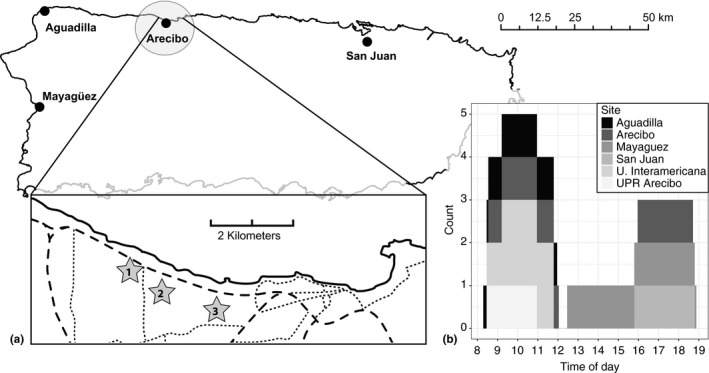
(a) Locations of sites sampled. Inset: in Arecibo, three locations were surveyed (indicated by stars): (1) University Ineteramericana Arecibo, (2) a residential neighborhood, and (3) University of Puerto Rico Arecibo. Dashed lines indicate major highways (thick dash) and major surface roads (small dash). (b) Distribution of hours sampled across all sites

From 24 April 2015 to 6 May 2015, we sampled six urban sites in four municipalities in northern and western Puerto Rico: San Juan, Arecibo, Aguadilla, and Mayagüez (Figure 2a). In San Juan, Aguadilla, and Mayagüez, the sites were residential neighborhoods. In Arecibo, we sampled one residential neighborhood and two university campuses (University of Puerto Rico Arecibo and University Interamericana). For logistical reasons, we did not sample nearby forested areas. Our visual sampling methods used in the urban habitat would likely have been inadequate in forest habitats due to the differences in structural habitat complexity and canopy use by *A. stratulus*.

At each locality, we sampled abundance by slowly walking through the habitat for a minimum of 3 hr without retracing our path between 8 a.m. and 7 p.m. (dusk). Because these species vary in their habitat use throughout the day in natural forest habitats (Hertz, 1992; Nicholson et al., 2005) and in urban habitats (Avilés‐Rodríguez *unpublished data*), surveys were conducted at different times of the day with at least one site surveyed during each daylight hour (Figure 2b). For every adult lizard observed, we recorded species and perch substrate.

We chose a subset of the observed lizards as focal animals. We only chose focal animals occupying unique perches to avoid accidental pseudoreplication (i.e., we did not sample multiple lizards of the same species utilizing the same tree). Because of differences in abundance, we sampled every *A. stratulus* occupying a unique perch and the next observed *A. cristatellus*, resulting in approximately equal numbers of focal observations per species. For focal animals, we noted environmental conditions and habitat use: ambient temperature (digital probe: OMEGA HH12B), perch temperature (infrared probe: EXTECH IR201), relative humidity (digital meter: AMPROBE THWD‐3), sun conditions, perch height, perch diameter, distance to nearest potential perch (any structure at least 0.5 m high and robust enough to support the weight of an adult of either species), perch type, and perch roughness. Perch roughness was rated on a scale of 1–5, with lower numbers indicating smoother substrates. Ratings were as follows (based on previous analyses of surface roughness and substrate type; Winchell et al., 2016): (1) glass, (2) metal, (3) painted concrete, (4) unpainted processed wood, leaves, and smooth bark trees such as bamboo, and (5) thick bark trees such as *Tababuya amarillo* and *Calophyllum antillanum* as well as unpainted and weathered concrete. We took photos of the canopy immediately above the perch site (Olympus EP5 with Olympus 9 mm 1:8.0 fisheye). We estimated vegetative canopy cover and manmade canopy cover (i.e., manmade structures obscuring the sky) from these photographs using Adobe Photoshop (CS5.1).

For every focal animal, we also sampled available habitat by randomly selecting a nearby perch. We sampled nearby available perches by choosing the closest potential perch in a random direction from the sampled perch. We collected the same habitat measurements as those noted above at three heights: 0.5, 1.5, and 2 m above the ground resulting in a set of three random perches for every utilized perch. For perches <2 m in height, we sampled only at 0.5 and 1.5 m. Wall perches greater than 50 cm in diameter were recorded as 100 cm as diameter cannot be sensibly calculated for completely flat perches (e.g., walls) and perches of this size are likely functionally equivalent for small lizards (e.g., Cartmill, 1985). We log‐transformed perch height, diameter, and distance to nearest perches before subsequent statistical analyses.

We analyzed differences in habitat choice and niche space with three main analyses. We performed all statistical analyses using R 3.2.2 (R Core Team 2015). First, we tested for differences in habitat utilized by each species compared to the randomly available habitat (i.e., habitat choice) by fitting a two‐way MANOVA for each species with site and sample number (to account for correlation between available habitat samples) as covariates (residuals for these models can be found in the supplemental materials S1‐S3). We used Fisher's Exact and Chi‐square tests (as appropriate) to compare perch type used between species and between each species and randomly available perches. We did not include perch height in this analysis as we did not randomly sample available perches at the full range of potential heights (e.g., above our reach). Second, we determined key habitat variables distinguishing perch occupancy between the two species using conditional inference classification tree analysis. This analysis determines predictive variables with the greatest explanatory power to separate groups and provides threshold estimates for each variable. We fit our classification model using the R package “party” (Hothorn, Hornik, & Zeileis, 2006). Finally, we analyzed how the two species partition the habitat as a whole with a varimax‐rotated principal component analysis on the correlation matrix for utilized and available habitat data using the variables for which habitat choice was suggested by the MANOVAs for at least one species. We retained components with eigenvalues >1. For retained components, we then compared multidimensional habitat use between species with *t* tests, and between utilized and available habitat with ANOVA and Tukey's HSD post hoc tests.

## 3 RESULTS

Collectively, we spent a total of 64 person‐hours at 5 urban sites and recorded 487 lizards. Of these, 442 were *A. cristatellus* (mean 6.68 lizards per person‐hour per site) and 45 were *A. stratulus* (mean 0.73 lizards per person‐hour per site). In addition, we sampled 31 lizards (*n* = 20 *A. cristatellus* and *n* = 11 *A. stratulus*) from a sixth site (Arecibo UI) in which we did not record total sightings. Of these 518 animals, we sampled a total of 47 *A. stratulus* and 52 *A. cristatellus* as focal animals for habitat use and took a total of 274 measures of potential perch sites (“available habitat”).

**Figure 3:**
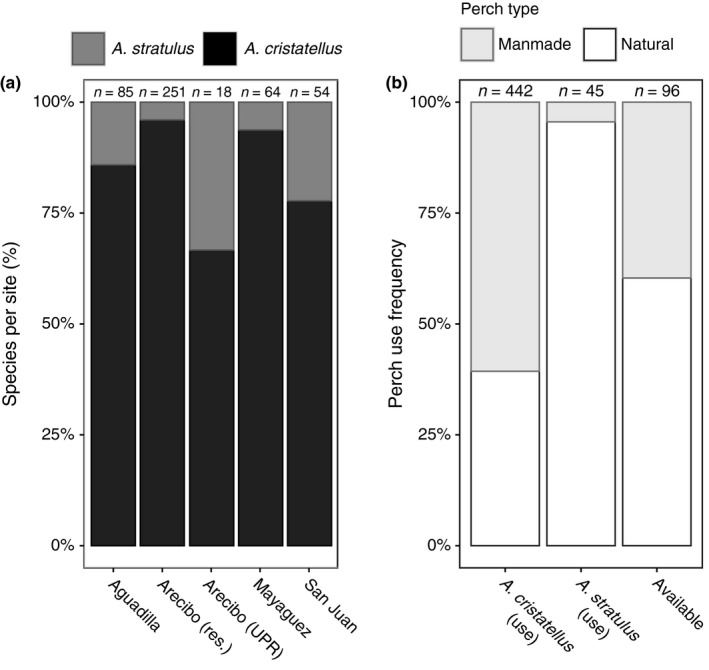
(a) Relative abundances differed dramatically: we encountered many more *A. cristatellus* across all sites. (b) Anolis cristatellus used artificial perches at a higher frequency than natural perches and at a similar rate to what was available. *Anolis stratulus* used natural perches almost exclusively (Fisher's Exact test, *p* < .001)

Assuming detection probability is similar for the two species, *A. cristatellus* were significantly more abundant than *A. stratulus* across sites (Fisher's Exact, *p* < .001; Figure 3a). Comparing all observed animals from the five sites in which we recorded total sightings, we found that *A. cristatellus* utilized manmade substrates such as walls and fences at a much higher rate (62%) than *A. stratulus* (4%) (Fisher's Exact, *p* < .001; Figure 3b). However, when comparing these observations to all random perch sites (*n* = 96), we found that *A. stratulus* uses natural substrates more frequently than encountered (Fisher's Exact, *p* < .001) while *A. cristatellus* uses manmade substrates at a relative frequency similar to their availability in the environment (χ^2^, *p* = .535).

**Figure 4:**
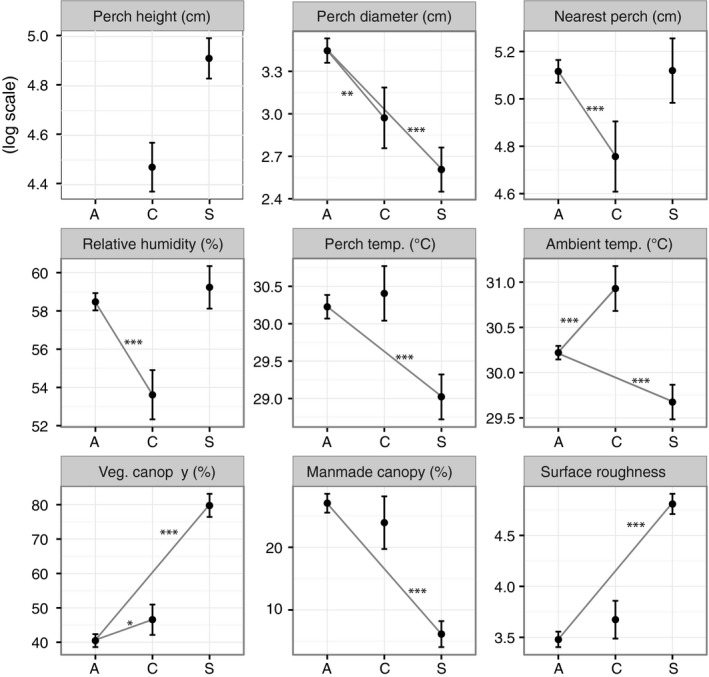
Mean and SE for habitat variables sampled for habitat availability (“A”), and each species use (“C”—*A. cristatellus*, “S”—*A. stratulus*). Differences between utilized and available habitat (summarized in Table 2) represented by gray lines and significance level: ***p < .001, **p < .01, *p < .05

We found that available habitat differed significantly from utilized habitat for *A. cristatellus* in five variables: ambient temperature, humidity, perch diameter, perch proximity, and vegetative canopy cover. In *A. stratulus*, available and utilized habitat differed for seven variables: ambient temperature, perch temperature, perch diameter, vegetative canopy, manmade canopy, perch type, and perch roughness (Tables 1, 2; Figure 4).

**Table 1:** Results from MANOVAs for habitat use versus availability for the nine habitat variables comparing the six urban sites and habitat choice. Degrees of freedom (*df*), *F*, and *p*‐value (*p*) are given for each

	Wilks’ lambda	*df*	*F*	*p*
*Anolis cristatellus*				
Habitat choice	0.569	1, 216	17.459	**<.001**
Urban site	0.025	5, 216	26.549	**<.001**
*Anolis stratulus*				
Habitat choice	0.247	1, 212	68.827	**<.001**
Urban site	0.010	5, 212	36.574	**<.001**

Statistically significant values for habitat choice are bolded and indicate differences between utilized and randomly available habitat.

**Table 2:** Results from ANCOVAs for habitat choice, subsequent to the MANOVAs in Table 1. Degrees of freedom (*df*), *F*, and *p*‐value (*p*) are given for each

	*df*	*F*	*p*
*Anolis cristatellus*			
Ambient temperature	1, 216	55.822	**<.001**
Perch temperature	1, 216	0.331	.566
Humidity	1, 216	99.237	**<.001**
Perch height	1, 216	5.868	**.016**
Perch diameter	1, 216	9.012	**.003**
Perch proximity	1, 216	23.067	**<.001**
Vegetative canopy	1, 216	4.028	**.046**
Manmade canopy	1, 216	1.437	.232
Perch roughness	1, 216	1.589	.209
*Anolis stratulus*			
Ambient temperature	1, 212	47.060	**<.001**
Perch temperature	1, 212	29.113	**<.001**
Humidity	1, 212	2.660	.104
Perch height	1, 212	0.197	.658
Perch diameter	1, 212	40.113	**<.001**
Perch proximity	1, 212	0.187	.666
Vegetative canopy	1, 212	319.157	**<.001**
Manmade canopy	1, 212	104.938	**<.001**
Perch roughness	1, 212	238.820	**<.001**

Shaded cells are significant for difference between use and availability at the significance level indicated by the last column.

In terms of structural habitat (Table 2, Figure 4), *A. cristatellus* perched lower than *A. stratulus* (mean perch height *A. cristatellus*: 1.080 m, mean perch height *A. stratulus*: 1.561 m; *t* = −3.293, *df* = 88, *p* = .001). Both species utilized thinner perches than are randomly available, which is unsurprising given the large number of extremely broad diameter perches in urban areas (e.g., walls). *Anolis cristatellus* also chose perches that were less isolated (i.e., perches that were closer to an alternate perch site), while *A. stratulus* did not discriminate on this axis. In addition, *A. cristatellus* did not discriminately use habitat based on perch type (manmade vs. natural) or substrate roughness, whereas *A. stratulus* used natural perches and perches that had rougher surfaces at a higher frequency than expected based on their availability.

In terms of microclimate (Table 2, Figure 4), both species actively selected microhabitat based on ambient temperature, though in opposite directions: *A. cristatellus* utilized warmer habitat while *A. stratulus* utilized cooler habitat. In addition, *A. cristatellus* used perches that had lower humidity, and *A. stratulus* used perches that had lower surface temperatures. Although both manmade (shade cast by built structures) and vegetative (shade cast by trees or other vegetation) canopy covers were low across all sites, *A. stratulus* discriminately utilized perch sites with very high vegetative canopy cover and very low manmade cover compared to random perches. *Anolis cristatellus* also chose perches with slightly higher vegetative canopy than expected by chance, but this effect was relatively weak and *A. cristatellus* did not appear to discriminate for or against manmade cover.

**Figure 5:**
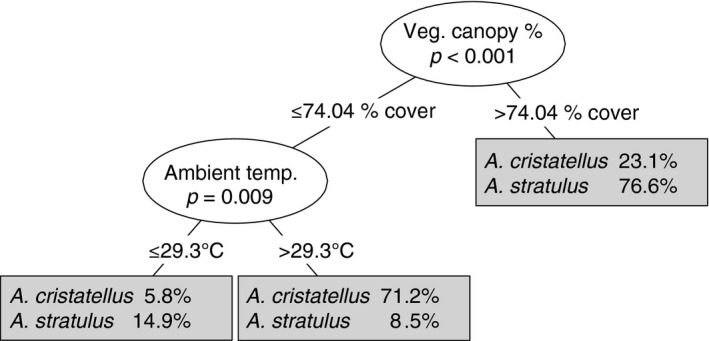
Classification tree for habitat use differences in *A. cristatellus* and *A. stratulus*. Species percentages indicate the percentage of all sampled individuals of each species in each group (high canopy cover, low canopy cover and high temperature, low canopy cover and low temperature)

We next analyzed species presence at a perch site using classification tree analysis to identify habitat factors that differentiate perch appropriateness between the two species (Figure 5). This analysis revealed that habitat use in *A. stratulus* and *A. cristatellus* separates based on two key variables. The species differed primarily in habitat use based on vegetative canopy cover (*p* < .001), with the majority of *A. stratulus* using perches covered by greater than 74.0% tree cover; and secondarily based on ambient temperature (*p* = .009) with the majority of *A. cristatellus* using habitat with lower canopy cover and ambient temperatures greater than 29.3°C.

**Figure 6:**
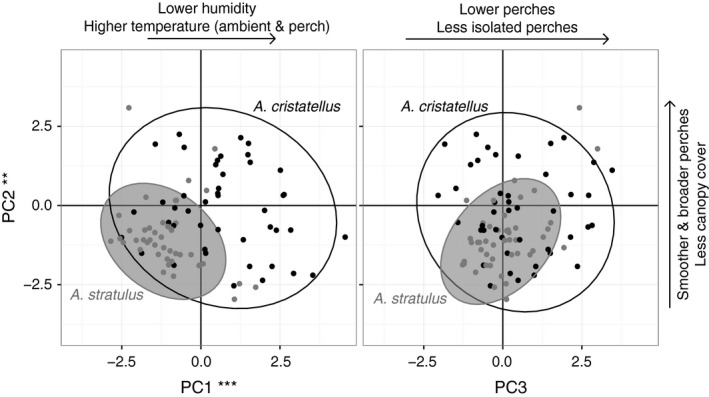
Principal components 1, 2, and 3 plotted with 95% confidence interval ellipses for utilized habitat by each species. PC1 and PC2 differed between species at significance levels *p* < .001 (***) and *p* < .01 (**), respectively

Lastly, we analyzed multi‐dimensional niche space with a varimax‐rotated principal component analysis of these habitat variables. We included perch height and seven of the eight significant variables from the MANOVA analyses in this analysis to determine differences between species in multivariate niche space. We included only one of the canopy cover variables (vegetative canopy) because we are interested in describing niche space without any explicitly urban variables included (e.g., manmade canopy cover). Principal components 1 and 2 were significantly different between *A. stratulus* and *A. cristatellus* (*t* test; Table 3, Figure 6), and the first three principal components captured 65.3% of variance. Varimax‐rotated component 1 had high positive loadings for ambient and perch (surface) temperatures and a high negative loading for humidity. Component 2 had high a positive loading for perch diameter and high negative loadings for vegetative canopy cover and perch roughness. Finally, component 3 had high negative loadings for perch height and proximity to nearest perch.

**Table 3:** Results from varimax‐rotated principle component analysis of urban habitat

	PC1	PC2	PC3
Humidity	**−0.560**	0.118	**−**0.163
Ambient temp.	**0.616**	**−**0.008	0.010
Perch height	**−**0.112	**−**0.040	**−0.571**
Perch diameter	**−**0.188	**0.477**	**−**0.048
Nearest perch	0.136	0.011	**−0.786**
Veg. canopy	**−**0.125	**−0.631**	0.014
Perch temp.	**0.473**	0.108	**−**0.136
Perch roughness	**−**0.042	**−0.589**	**−**0.087
Cumulative % variance	26.87	52.05	65.28
Significance: Species	*p* < .001***	*p* = .001**	*p* = .107
Significance: perch type	*p* < .001***	*p* < .001**	*p* = .002**
Eigenvalue	2.150	2.015	1.058

Significance for *t* tests comparing principal components between *A. stratulus* and *A. cristatellus* are given in the row labelled “Significance: Species,” and significance for *t* tests comparing principal components between natural and manmade perches are given in the row labelled “Significance: Perch Type”. Significant loadings are bolded and shaded. Significance levels for species and perch type ANOVAs are: ***p < .001, **p < .01.

**Figure 7:**
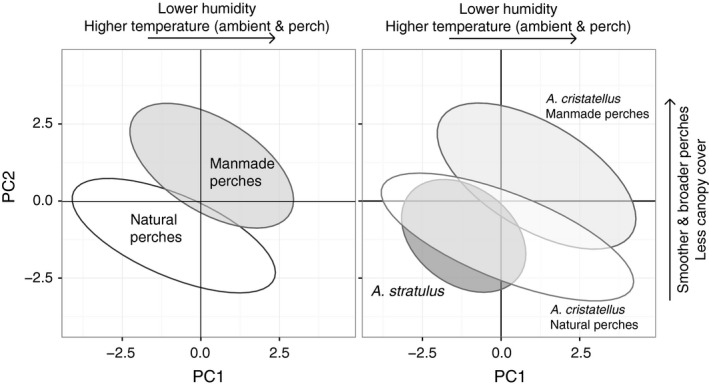
Principal components 1 and 2 plotted with 95% confidence interval ellipses grouped by perch type for available habitat (left) and utilized habitat (right)

We anticipated that many of the relevant environmental factors could be associated with artificial substrates, so we also compared multidimensional niche space of available and utilized perches associated with natural and artificial substrates (Figure 7). Available habitat differed between artificial and natural perch types for all three principal components, suggesting that habitat associated with manmade perches differs from that associated with natural perches in multiple dimensions (Figure 7; Table 3). Finally, we compared utilized habitat separated by perch type for *A. cristatellus* to utilized habitat for *A. stratulus*. We found that manmade perches used by *A. cristatellus* differed from natural perches used by *A. cristatellus* and from all perches used by *A. stratulus* for PC1 and PC2 (Figure 7; Table 4), suggesting that divergence in habitat use between the species is associated with the use of manmade perches.

**Table 4:** Results of ANOVA and Tukey's HSD comparing principal components between utilized habitat by *A. stratulus* (all) and *A. cristatellus* (by perch type). Degrees of freedom (*df*), *F*, and *p*‐value (*p*) are given for each test

	ANOVA			Tukey's HSD		
	*df*	*F*	*p*	CN–CM	CN–S	CM–S
PC1	2, 86	24.970	**<.001** ***	**.0313** *	**.001** ***	**<.001** ***
PC2	2, 86	28.890	**<.001** ***	**<.001** ***	.695	**<.001** ***
PC3	2, 86	1.323	.272	1.000	.413	.361

Paired comparisons for the Tukey's HSD tests are *A. cristatellus* using natural perches versus *A. cristatellus* using manmade perches (CN–CM), *A. cristatellus* using natural perches versus *A. stratulus* on all perches (CN–S), and *A. cristatellus* using manmade perches versus *A. stratulus* on all perches (CM–S). Significance levels: ***p < .001, **p < .01 , *p < .05; Significant results are bolded and shaded.

## 4 DISCUSSION

### 4.1 Abundance

We found that *A. cristatellus* was significantly more abundant than *A. stratulus* at sampled urban sites. In particular, we encountered nearly 10 times as many *A. cristatellus* as *A. stratulus* across all sites, and the former species was encountered at a much higher rate per person‐hour. This finding could simply be a consequence of differences in abundance in natural habitats nearby. Both *A. cristatellus* and *A. stratulus* are locally abundant in natural habitats in Puerto Rico with sympatric densities in mesic habitats at ground level estimated at 1,000–1,100 and 600–900 per hectare, respectively (Rodda, Perry, Rondeau, & Lazell, 2001). If fewer *A. stratulus* are found in nearby natural areas, then we might expect urban habitats to inherit this natural difference in abundance between species. However, the difference in abundances measured in the present study far exceeds that found previously for Puerto Rican mesic forest (e.g., Rodda et al., 2001). This suggests that urban habitats do not support *A. stratulus* and *A. cristatellus* in relative abundances proportional to those reported for more natural sites. Relative abundances in urban areas instead appear to follow a pattern more closely resembling naturally xeric forests (e.g., Genet, Genet, Burton, & Murphy, 2001), despite being located in mesic regions.

It is possible that our sampling method may have failed to detect *A. stratulus* perching higher in the urban canopy. Indeed, in natural habitats, *A. stratulus* density has been underestimated at some sites when they utilize canopy habitat. For example, Reagan (1992) found that *A. stratulus* attains extremely high densities (24,000–28,000/ha) in forests where individuals occupy high canopy habitat (approximately 10–20 m height). We believe this is not a major concern in our study as urban trees at our sites tended to be shorter and rarely produced dense or continuous canopy. Moreover, manmade substrates such as walls and fences are clearly visible all the way to the roofline. As such, visibility of the few high perches present was very good across all urban sites and we do not expect that we failed to observe a significant number of individuals at higher perches.

### 4.2 Habitat use

The two lizard species of this study differed in the frequency which they utilized manmade substrates as perches. *Anolis cristatellus* used manmade perches at a high frequency, but used both manmade structures and vegetation in proportion to their relative abundance in the environment. In contrast, *A. stratulus* infrequently occupied manmade perches. Similarly, Kolbe, Battles, and Avilés‐Rodríguez (2015), found that *A. cristatellus* used manmade structures more frequently than *A. stratulus* in human‐modified habitats, despite finding *A. stratulus* to be more adept at sprinting on smooth vertical surfaces. Our data suggest that *A. stratulus* either actively seeks out natural substrates or avoids the use of manmade substrates. This pattern may be explained by some combination of predation, competition, performance, and habitat requirements.

At first glance, it appears that *A. cristatellus* and *A. stratulus* partition urban niche space in similar ways as in natural forest habitat. When found syntopically in natural forest habitat, these two species divide the habitat, such as many anole species, based primarily on structural and microclimatic habitat features (Rand, 1964; reviewed in Losos, 2009). In natural habitats, *A. cristatellus* typically uses broader and lower perches while *A. stratulus* uses thinner and higher perches (Cooper, 2005; Rand, 1964; Reagan, 1992). The complexity of the perches chosen also differs, with *A. cristatellus* using relatively simpler, less branching perches compared to *A. stratulus* (Powell & Leal, 2014). In terms of climatic habitat, in natural areas, *A. cristatellus* typically uses perches that are higher in temperature and lower in humidity, often with reduced tree cover, compared to those used by *A. stratulus* (Cooper, 2006; Heatwole, Lin, Villalón, Muñiz, & Matta, 1969; Rand, 1964).

In urban habitats, we found that the two species select structural habitat based on similar factors. In particular, we found that *A. cristatellus* utilized broad and low perches while *A. stratulus* utilized thin and high perches. Reagan (1992) found higher perches also tend to be thinner and concluded that perch diameter, not height, may be the driving factor in determining structural habitat use in *A. stratulus*, perhaps because larger predatory species cannot locomote effectively on thinner perches. In addition, *A. stratulus* is thought to more strongly rely on crypsis to avoid predation while *A. cristatellus* more often flees from potential predators (Cooper, 2006; Heatwole, 1968). If *A. stratulus* experiences elevated predation on substrates where it is more exposed, or if it is unable to compete with the larger and predatory *A. cristatellus* on these substrates, it may avoid this habitat type. These factors could help explain the pattern of habitat use that we observed in urban settings. In particular, the majority of manmade perches in urban areas are broad and simple (i.e., non‐branching) and differ in pattern and color compared to natural substrates. Thus, these surfaces offer relatively few refuges, little opportunity to avoid predators via crypsis, and no locomotor advantages for species adapted to thinner perches, such as *A. stratulus*.

We also found that both species segregate urban habitat based on microclimatic variables similar to those in natural habitats. *Anolis cristatellus* selected perches with higher temperature, lower humidity, and relatively little canopy cover, while *A. stratulus* chose perches with lower surface and air temperatures, and more extensive canopy cover. Differences in thermal preferences may be key to understanding both the lower abundances and preference for heavily shaded natural substrates that we show for *A. stratulus*. Specifically, the microclimatic factors on which this species discriminately chooses perches are simply less common in urban environments. The urban habitat tends to be hotter and drier due to increased impervious surface and decreased canopy cover, and manmade substrates have different thermal properties compared to natural surfaces (Forman, 2014). Studies from natural forest habitats suggest that these species differ in thermal physiology. For example, juvenile *A. stratulus* in xeric forests are sensitive to low humidity and high temperature conditions (Nicholson et al., 2005) and adult *A. cristatellus* from both lowland and upland mesic forests prefer warmer temperatures and can tolerate higher temperatures compared to *A. stratulus* (Heatwole et al., 1969). *Anolis stratulus* may simply be too constrained to tolerate the physiological stresses associated with the most intensely built components of urban habitats.

### 4.3 Niche partitioning

Our classification tree analysis indicates that two environmental factors in particular are most important in determining habitat use: vegetative canopy cover and ambient temperature at perch sites. The majority of *A. stratulus* occupied perches with extremely high canopy cover, and the few that did not choose sites with relatively low ambient temperatures. In contrast, the majority of *A. cristatellus* occupied sites with both low canopy cover and high ambient temperatures. This indicates that thermal preferences and tolerances are likely a major factor in determining habitat use in urban areas. Interestingly, *A. stratulus* in this study chose perches that had high vegetative canopy cover but low manmade canopy cover. This suggests that some factor correlated with vegetative canopy cover (and not simply the lowered temperatures offered by shade) may be important in habitat use for this species, perhaps related to associated prey insects or microclimatic conditions.

The patterns of habitat use and availability paint an interesting picture of habitat partitioning in the urban environment. Both species demonstrate nonrandom habitat selection for different aspects of their structural and microclimatic environments. Our analysis of principal components shows that both structural and climatic attributes of the perch are significant in describing habitat use differences. Overall, *A. cristatellus* was more generalized in its habitat use while *A. stratulus* was highly selective, using a subset of both total available habitat and of the habitat utilized by *A. cristatellus*. Interestingly, although no explicitly urban habitat variables were included in the principal component analysis, the habitat space uniquely occupied by *A. cristatellus* was associated with characteristics of more intense urbanization: lower humidity, higher temperatures, smoother and broader perches, and decreased vegetative canopy cover. Use of this urban‐associated habitat space reduces niche overlap with *A. stratulus*, which is mainly restricted to habitat associated with natural perches. Whether this represents a preference for anthropogenic habitat by *A. cristatellus* or simply a tolerance of this abundant habitat type in urban areas is not a question we can answer with our current dataset, but is one that we hope to further explore in ongoing research.

In conclusion, we have shown that two common species of anoles that obtain high abundances in sympatry in natural habitats have very different responses to urbanization in terms of relative abundance and habitat use. The differential use of urban habitat in some ways reflects patterns observed in natural habitats in terms of structural and microclimatic habitat, but also demonstrates that these species are utilizing anthropogenic resources, specifically habitat associated with manmade perches, to differing degrees. We find that *A. stratulus* is restricted to natural habitat elements within the urban matrix while *A. cristatellus* exploits novel habitat associated with manmade structures. This division of the urban habitat results in decreased niche overlap associated with two distinct niche spaces: natural and manmade substrates. This provides empirical evidence for the hypothesis that some species are urbanophilic while others are merely tolerant of urbanization.

The Caribbean islands have experienced urbanization pressures for over five centuries, yet the implications of this habitat modification are only now being studied (e.g., Ackley, Muelleman, Carter, Henderson, & Powell, 2009; Germano, Sander, Henderson, & Powell, 2003; Mallery, Marcum, Powell, Parmerlee, & Henderson, 2003; Marnocha, Pollinger, & Smith, 2011; Winchell et al., 2016). Understanding the factors of urbanization to which species are able to adapt is critical to successful conservation management in urban habitats (Donihue & Lambert, 2015). Our findings reinforce the idea that understanding species’ natural history, particularly differences in habitat use, is an important precursor to understanding how species will respond to intensifying urbanization in coming years. Broad habitat preferences and a high frequency of utilization of urban resources, as we observed in *A. cristatellus*, set the stage for evolutionary adaptation to urban environmental change in urbanophilic species (e.g., Winchell et al., 2016). By contrast, apparent avoidance of urban substrates and a high frequency of utilization of the remnant natural resources, as we observed in *A. stratulus*, reminds us not to expect that all urban‐tolerant species will be equally resistant to local extirpation with increasingly intense urban development.

## CONFLICT OF INTEREST

None delcared.

## AUTHOR CONTRIBUTIONS

KMW and LJR conceived the project and designed methodology. KMW, ARPR, and LJR identified study locations and secured site permissions. KMW and EJC collected the data. KMW and LJR analyzed the data with input from ARPR and EJC. All authors contributed to the writing of the manuscript and gave final approval for publication.

## DATA ACCESSIBILITY

Data are available on Dryad Digital Repository (https://doi.org/10.5061/dryad.6fq63).
