# Supplementary material for: Divergent habitat use of two urban lizard species
Source: Ecol Evol. 2017 Nov 23;8(1):25–35. doi: 10.1002/ece3.3600 (PMC5756862; doi:10.1002/ece3.3600)
Supplement: Supplementary file 1 [file ECE3-8-25-s001.pdf]

DIVERGENT HABITAT USE OF TWO URBAN LIZARD SPECIES

Supplementary Materials

We assessed the residuals of each variable included in the MANOVA for normality using the Kolmogorov-Smirnov test. Residuals were non-normal within the three main groups (available habitat, *Anolis cristatellus* use, and *Anolis stratulus* use). However, the distributions of residuals for all variables were approximately normal with no bimodality, noticeable skew, or major outliers. We note that the KS test is sensitive and that OLS estimators are reasonably robust to minor deviations from normality. We provide the histograms of the residuals here.

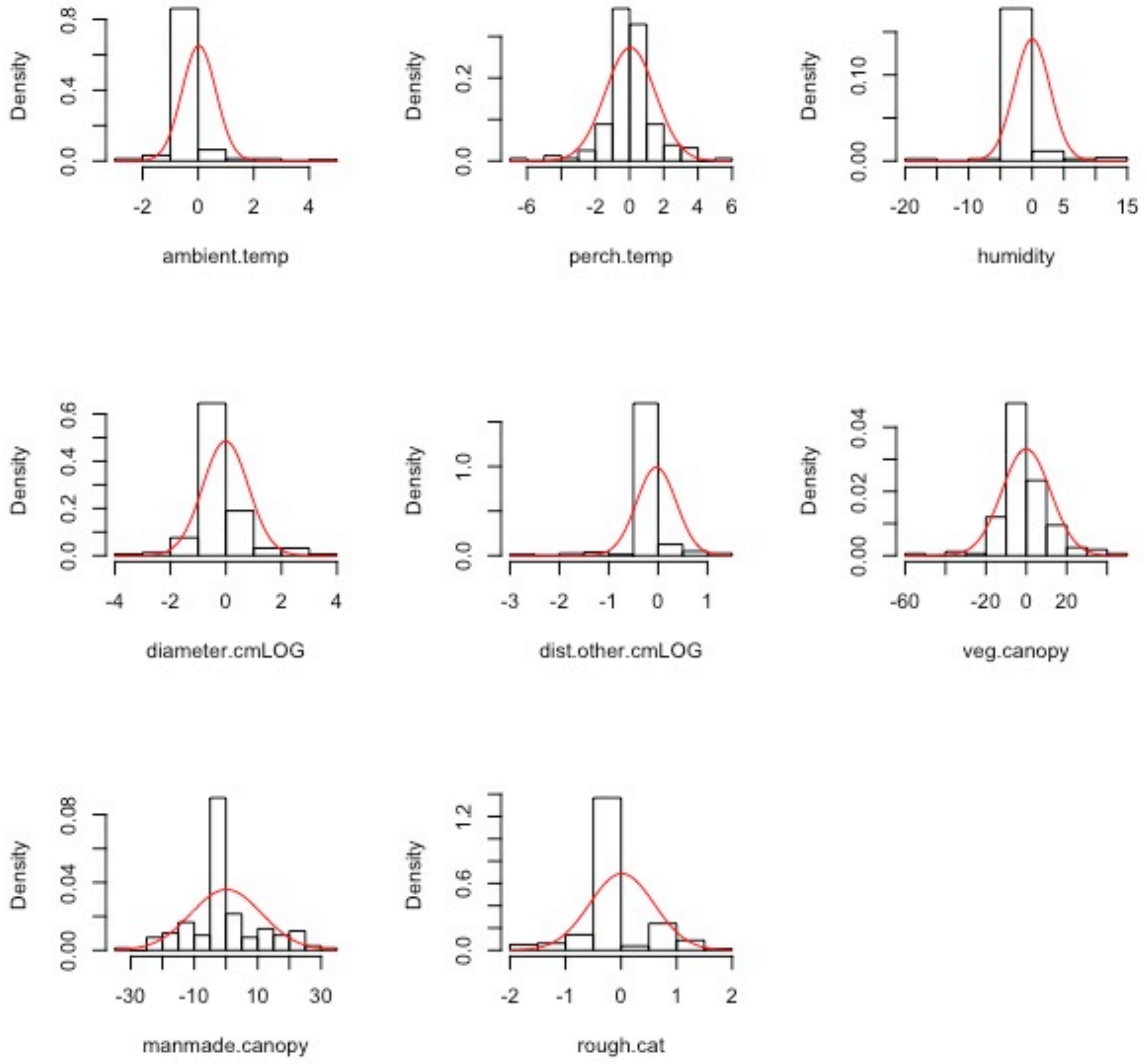

Figure S1. Residuals for each variable in the MANOVA for available habitat group.

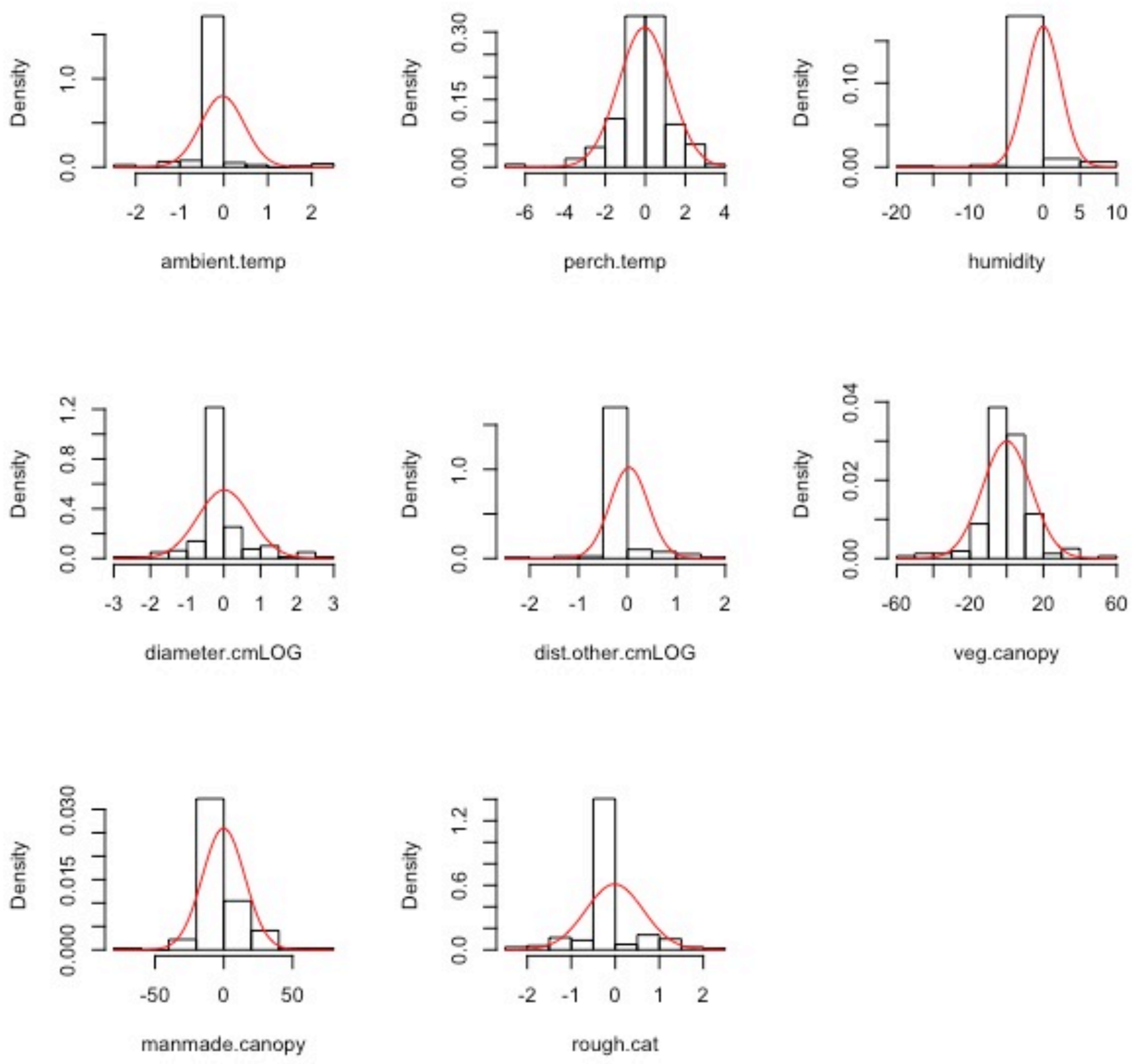

Figure S2. Residuals for each variable in the MANOVA for the A. cristatellus *utilized habitat group.*

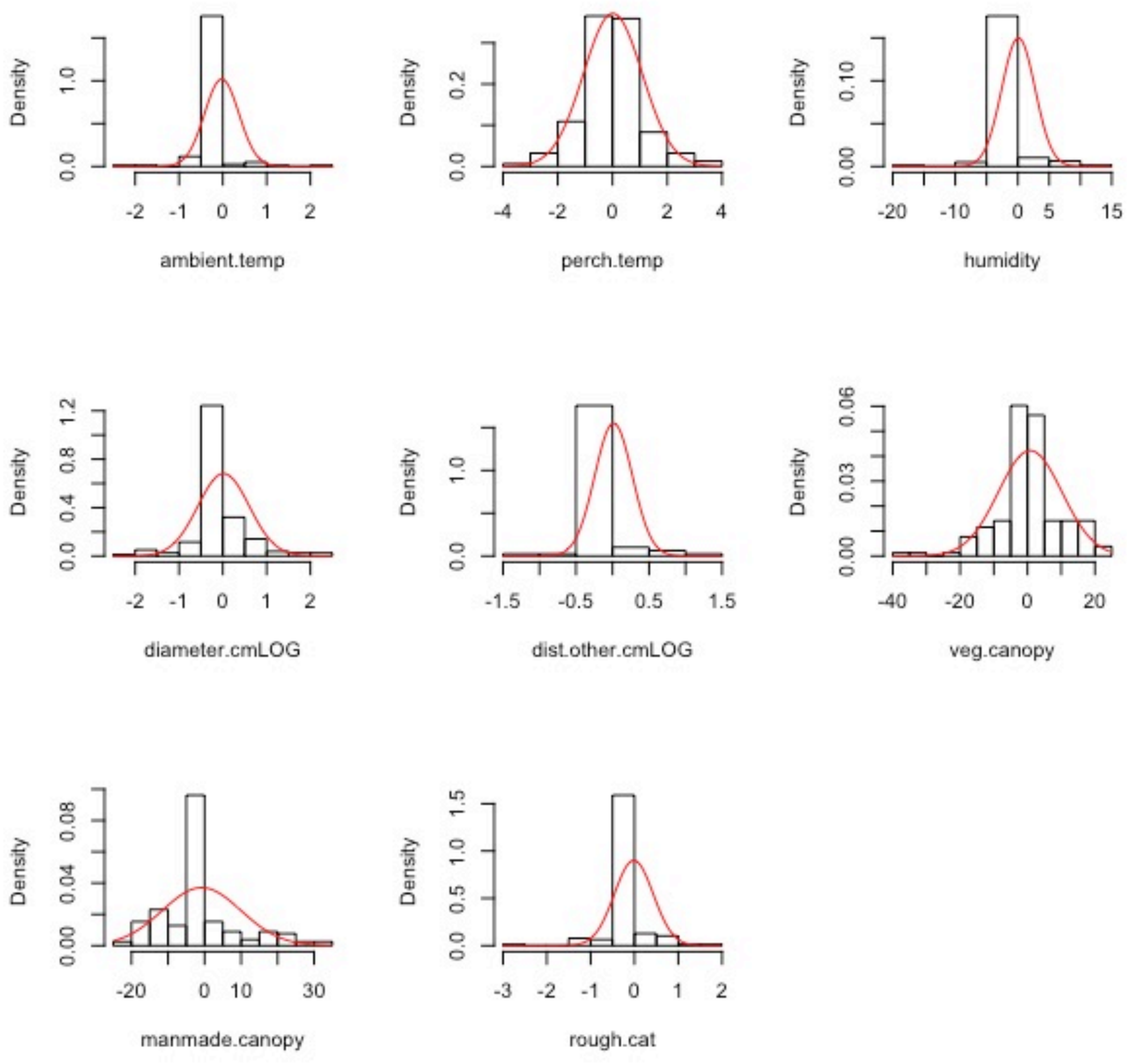

Figure S3. Residuals for each variable in the MANOVA for the A. stratulus *utilized habitat group.*
